# Developing the BornFyne prenatal management system version 2.0: a mixed method community participatory approach to digital health for reproductive maternal health

**DOI:** 10.1093/oodh/oqae012

**Published:** 2024-03-07

**Authors:** Miriam Nkangu, Mildred Nkeng Njoache, Pamela Obegu, Franck Wanda, Ngo Valery Ngo, Arone Fantaye, Mwenya Kasonde, Amos Wung Buh, Regina Sinsai, Evrard Kepgang, Odette Kibu, Sarah Pascale Ngassa Detchaptche, Nkengfac Fobellah, Ronald Gobina, Brice Tangang, Denis Foretia, Arthur Pessa, Julian Little, Donald Weledji, Sanni Yaya

**Affiliations:** School of Epidemiology and Public Health, University of Ottawa, Ottawa, Canada; Bruyere Research Institute, Ottawa, Canada; Health Promotion Alliance of Cameroon (HPAC), Yaounde, Cameroon; Health Promotion Alliance of Cameroon (HPAC), Yaounde, Cameroon; Health Promotion Alliance of Cameroon (HPAC), Yaounde, Cameroon; The International Centre for Research, Education and care (CIRES), Akonolinga, Cameroon; Denis and Lenora Foretia Foundation, Health Division Simbock, Nkafu Policy Institute, Yaounde, Cameroon; Education, Faculty of Medicine, University of Ottawa, Ottawa, Canada; Global Health, Liverpool School of Tropical Medicine, Liverpool UK; Population Health, Interdisciplinary School of Health Sciences, University of Ottawa, Ottawa, Canada; Denis and Lenora Foretia Foundation, Health Division Simbock, Nkafu Policy Institute, Yaounde, Cameroon; Denis and Lenora Foretia Foundation, Health Division Simbock, Nkafu Policy Institute, Yaounde, Cameroon; Denis and Lenora Foretia Foundation, Health Division Simbock, Nkafu Policy Institute, Yaounde, Cameroon; Public Health, University of Buea, Buea, Cameroon; Health Promotion Alliance of Cameroon (HPAC), Yaounde, Cameroon; Department of Family Health, Ministry of Public Health, Yaounde, Cameroon; Denis and Lenora Foretia Foundation, Health Division Simbock, Nkafu Policy Institute, Yaounde, Cameroon; IT Department, Donwel Systems Brussels, Brussels Belgium; Denis and Lenora Foretia Foundation, Health Division Simbock, Nkafu Policy Institute, Yaounde, Cameroon; Center for Multicultural and Global Health, University of Tennessee Health Science Center, Memphis, USA; Denis and Lenora Foretia Foundation, Health Division Simbock, Nkafu Policy Institute, Yaounde, Cameroon; School of Epidemiology and Public Health, University of Ottawa, Ottawa, Canada; IT Department, Donwel Systems Brussels, Brussels Belgium; School of International Development and Global Studies, University of Ottawa, Ottawa, Canada

**Keywords:** digital health, reproductive health, health systems, health equity, rural areas, maternal health

## Abstract

Despite the growing number of global initiatives aimed at reducing adverse maternal health outcomes, there remain critical gaps and disparities in access to maternal health services in Cameroon and across the sub-Saharan Africa. Digital health innovations represent unique opportunities for addressing maternal and newborn child health in sub-Saharan Africa. This article documents the approach to developing the BornFyne-Prenatal Management System (PNMS) as an intervention to support maternal health issues in Cameroon. The mixed-method design employed the three-delays model conducted in four health districts purposefully selected with a mix of urban and rural settings as defined in the context. The study employed focus group discussions and interviews to inform the development features. A total of 25 providers were interviewed, 12 focus group discussions and 4 workshops were held and a total of 3654 households were surveyed. Participants highlighted multifaceted advantages of using digital health platform such as BornFyne-PNMS to enhance communication and care during pregnancy such as remote consultations, emergency response, increased patient engagement and improved continuity of care and convenience. Most respondents believed that the use of a digital platform like BornFyne-PNMS would greatly facilitate access to health facilities, especially during emergencies. The BornFyne-PNMS deployment includes community engagement, training and practical skills building of health workers in the use of digital technologies, the establishment of an emergency transport mechanism for response to emergency cases, assessment and upgrading of the computer hardware of enrolled health facilities and support to health system managers to review and interpret the BornFyne data and interoperability with the national health management information system.

## Teaser

In Cameroon and most parts of sub-Saharan Africa, there are significant challenges to accessing maternal health services. Despite global efforts, disparities persist especially in rural areas. Digital health platforms can help mitigate some of these challenges. This paper presents the process in the development of the BornFyne-PNMS platform in Cameroon. Through surveys, interviews, focus group discussions and workshops, potential users of the BornFyne-PNMS platform reported benefits of using a digital platform as BornFyne-PNMS to include improved communication, remote consultations, better emergency response network, increased patient engagement and enhanced continuity of care. The deployment of the BornFyne-PNMS platform involves community engagement, training of health workers, establishing emergency transport mechanisms, upgrading computer hardware in health facilities and supporting health system managers in interpreting and integrating BornFyne-PNMS data with national health information systems. This initiative aims to bridge critical gaps and improve maternal health outcomes in rural settings.

## INTRODUCTION

Despite the growing number of global initiatives aimed at reducing adverse maternal health outcomes, there remain critical gaps and disparities in access to maternal health services in Cameroon and across the sub-Saharan Africa (SSA) [1–17]. Digital health innovations represent unique opportunities for addressing maternal and newborn child health in SSA [11, 13]. This paper documents the approach to developing the BornFyne-Prenatal Management System (PNMS) as an intervention to support maternal health issues in Cameroon.

In the last 20 years, several global initiatives have been developed to help fast-track progress towards maternal health targets set in the millennium development goals and sustainable development goals [1–4]. The current literature shows barriers to access and utilization of routine and emergency maternal health services in SSA [1–17]. This includes physical, topographical and financial barriers and lack of knowledge, trust and awareness of available services. Consequently, the SSA region accounts for the highest burden of maternal deaths in the world [1–17].

Maternal health remains a public health emergency in Cameroon with a maternal mortality rate of up to 596 deaths/100 000 live births [2–10, 17, 18]. This has been attributed partly to the three delays [19]; delays in *seeking* care due to underlying social determinants of health, e.g. lack of knowledge on pregnancy, lack of education and gender inequality; delays in *reaching* care due to distance and cost and delays in *receiving* appropriate care, including poor quality of care at health facilities, high out-of-pocket expenses and poor adherence to clinical protocols [19, 20]. In addition to poor emergency and referral services, and long walking distances for women to reach motorable roads to access health facilities. Over the years, there has been a shift from the first and second delays to the third delay, recognized as a major contributor to maternal mortality and the most significant delay for women who died in the facility or at home after receiving care at the facility [21, 22]. For example, in 2016, an incident which has been attributed more to the third delay occurred in front of a health facility in Cameroon which led to the death of a woman and her unborn twin [23]. To propose interventions, it is crucial to recognize the specific factors that contribute to each delay, as they vary and play a significant role in decreasing maternal mortality. By identifying the factors that contribute to maternal mortality and designing interventions aimed at addressing these factors, we can work towards reducing maternal mortality rates and improving maternal health outcomes.

Some SSA countries have seen some progress of up to 45% reduction in the maternal mortality ratio between 1990 and 2015 [9], with varied improvements in access to antenatal, childbirth and postnatal care services [10–12]. This progress is partly attributable to transformative technologies [9–12]. However, there are still critical gaps in access and utilization especially in antenatal care (ANC), family planning and skilled birth deliveries [13–16]. In an attempt to further address some of the delays in seeking, accessing and receiving maternal care services, the BornFyne-PNMS, an innovative digital health solution was developed and tested in 2018 in the Northwest region of Cameroon [24]. The first phase was implemented in 2018–2019 and assessed acceptability and feasibility in an ideal setting with 140 poor pregnant women (PW) in Bali health district, in Northwestern Cameroon, in two health facilities [24, 25]. The initial assessment showed that the digital solution was acceptable to users and feasible to implement but needed to include the provision of ANC and post-natal care (PNC) vouchers (to remove out-of-pocket payments), smartphone devices and solar chargers and computer hardware and solar charging systems to health facilities [25] for ease of implementation. The second phase of BornFyne’s proof-of-concept is currently being implemented in four districts (both French and English-speaking) to assess BornFyne’s acceptability and feasibility in a real-world setting, without the provision of e-vouchers or smartphone devices and solar chargers [26].

In 2020, the Ministry of Public Health of Cameroon launched its digital health framework [20]. This framework identifies many challenges facing the health system in Cameroon, as highlighted by the Ministry of Public Health. They include geographic inaccessibility, low demand for services, delay in care delivery, poor adherence to clinical protocols and high out-of-pocket expenses—which to some extent may be mitigated through the contribution of digital health interventions [20]. The framework outlines the importance of health facilities being digitalized, and some of the main objectives listed are the need to train and build the capacity of health providers and health facilities to use digital platforms, deliver maternal care, deploy existing applications within current structures, develop interoperability layers with the existing district health information system (dhis2) and overall transformation and development of national infrastructures to support digital health platforms [20].

In 2021, the World Health Organization (WHO) launched the WHO Digital Adaptation Kit (DAK) for ANC as part of the Standards-based, Machine-readable, Adaptive, Requirements-based and Testable (SMART) guidelines which constitute a package of business process workflows, core data elements, decision-support algorithms, linkages to indicators and functional requirements that are more easily translated into digital systems [27–30]. This article therefore documents the approach to developing the BornFyne-PNMS, as an intervention to support maternal health issues in Cameroon. The article discusses the underlying assumptions and framework that informed the development of BornFyne-PNMS version 2.0 refined to integrate the WHO DAK for the ANC features and community user feedback from the BornFyne-PNMS version 1.0.

### BornFyne prenatal management system (PNMS) version 2.0

**Figure 1:**
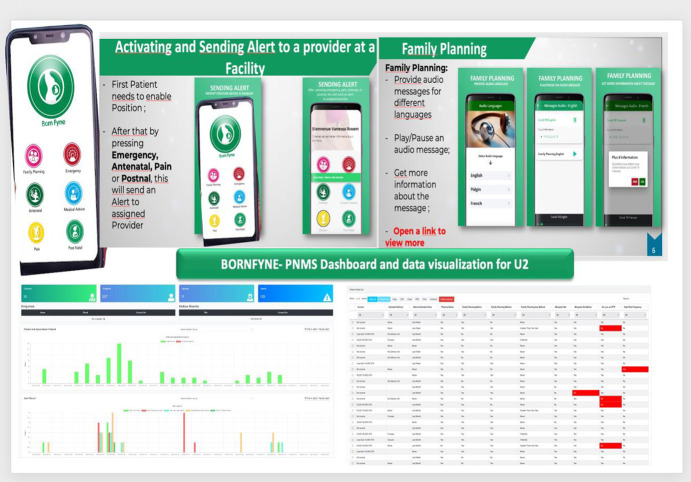
BornFyne-PNMS version 2.0

BornFyne-PNMS is a digital health solution that targets two distinct sets of users: PW and households defined as user 1 (U1) and health care providers and health facilities defined as user 2 (U2) [24–26]. The BornFyne-PNMS platform includes (i) A client-facing smartphone application designed with a graphical interface for easy navigation by women with low literacy. It includes six modules (see Fig. 1): two modules function entirely offline and allow the user to listen to pre-recorded audio content in the local dialect on family planning and other priority health topics [26]. The two offline modules allow users to engage with educational health content on family planning, malaria, COVID-19, ANC/PNC and any other locally relevant health information [26]. The innovation uses prompts and cues to trigger behavior change using both the user interface and the web-based interface. These user-friendly pictographs are designed using a community engagement approach to ensure that they reflect community perspectives and local context. This serves as a continuous educational platform for women and households in the comfort of their homes compared to standard practice which only relies on sporadic campaigns and during ANC visits. The content is developed following extensive user engagement to identify priority topics knowledge gaps and user concerns. These messages are audio-recorded and uploaded in local spoken languages in addition to supplemental links for those who can read in English and French.

The four online modules allow the user to receive personalized ANC and PNC reminder messages (according to their unique medical profile) and contact a health worker for triage and linkage to emergency transport services in the case of an emergency [24, 26] (U1). Four modules require network access to connect to the facility (see fig. 1). These include the ANC and postnatal modules, and two modules that allow a mother to request emergency support in the case of pain or a medical emergency.

(ii) A health worker-facing electronic medical record that structures data entry during antenatal and postnatal visits for more complete, accurate and timely data collection. The ANC module has been designed by integrating the WHO DAK for ANC guidelines [27, 30] to support health workers in their adherence to protocols and clinical decision-making (U2). Also, the U2 interface is structured to facilitate interoperability with existing health management information systems to enhance the quality-of-care delivery and improve data in an accessible and actionable format to inform policies and program interventions strategies towards reducing maternal mortality and achieving universal health coverage.

## METHODOLOGY

The three-delays model has been widely used to assess and understand maternal mortality in different contexts [19, 21, 22]. The model was used to inform the development of the BornFyne-PNMS digital platform to address maternal health issues and the underlying features of the BornFyne-PNMS platforms.

### Setting and context

Cameroon is a lower middle-income country, with a population estimate of 26 million in 2020 [18]. Women comprise up to 50% of the population [18, 31, 32]. Health is financed mainly through out-of-pocket expenditure [32]. Cameroon is made up of 10 regions, 2 of the regions are English speaking and 8 are French speaking. There are two official national languages, French and English and over 250 locally spoken dialects. The selection of the four districts for this study aimed to encompass linguistic and regional diversity in Cameroon, reflecting the dual linguistic context of the country. Two French-speaking districts (Akonolinga and Ayos) and two English-speaking districts (Tiko and Bangem) were chosen purposefully to ensure a comprehensive representation of rural areas. The goal was to capture potential variations in healthcare practices, cultural factors and health outcomes associated with linguistic differences. While higher levels of adolescent pregnancy were noted in French districts later in the paper, it’s important to clarify that the selection was primarily driven by the need for a balanced and inclusive examination of ANC accessibility across distinct linguistic and regional contexts in rural Cameroon. Based on information gathered from the district, the four districts Tiko, Bangem, Akonolinga and Ayos represent a total population of 164 284, 26 480, 90 565, and 44 153, respectively.

In Cameroon, up to 71% of the population live below $5 per day and over 50% live under $2 per day [18, 31, 32]. Up to 44% of the population live in rural settings [18, 31–34]. In addition, most women only tend to learn about family planning during antenatal or postpartum clinic visits, in which some are unable to adequately access due to high out-of-pocket expenses [34]. In 2017, the country adopted a performance-based financing program that pays provider incentives based on predefined quality and quantity criteria as a national strategy to improve the quality-of-care delivery for RMNCH and health providers’ performance [33]. Recently, the country has launched a universal health coverage program which is currently being pilot tested in selected districts. The health system is decentralized with a pyramidal structure that includes the national where the polices are defined, the regional level where the policies are translated into operations and the operational level which is the district where the policies are operational into actions [20, 33]. Healthcare is delivered in both private and public sector. The private sector is made up of private for profit and confessional health facilities (this includes faith based Catholic, Baptist and Presbyterian). In rural Cameroon, healthcare is provided through a mix of private, public and confessional facilities. The distribution and types of these facilities impact access to healthcare in the study districts, with private establishments, government-run facilities and those affiliated with religious organizations all playing significant roles. This diversity impacts the healthcare landscape and is essential to understand ANC for PW in the studied areas.

The rural health system in Cameroon comprises primary healthcare centers, community health posts and district hospitals, providing essential services at local and regional levels. These structures are overseen by the Ministry of Public Health in collaboration with regional health delegations, forming an integrated national health system. This setup ensures a continuum of care, effective resource allocation and communication channels between local and national levels.

### Study design

This is a mixed-method design and part of a larger study. The formative study employed participatory action research approach through focus group discussions (FGDs), workshop and interviews to inform the development features. Drawing from lessons learnt and user feedback from the initial pilot in Bali district. In addition, a household survey was initially conducted to explore the community’s perspective on introducing a digital platform that connects PW or households to a health facility as an intervention mechanism to address the delays in accessing and receiving care during pregnancy.

**Figure 2:**
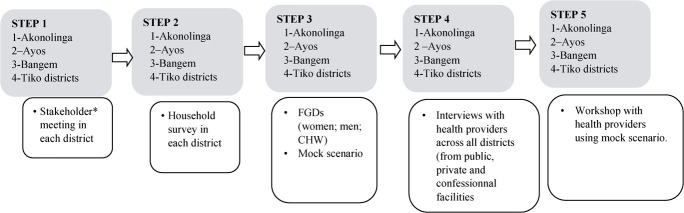
Sampling of household

The development and refinement of the BornFyne-PNMS were informed by the three-delays model using a community participatory action research approach and engagement strategies through FGDs, interviews, surveys and workshops to ensure acceptability, usability and adaptability (see Fig. 2). This approach is ideal for co-developing interventions with the people rather than for the people [35]. In addition, stakeholder meetings were organized in all four districts including the regional and central level [26]. The stakeholder meetings were part of the community mobilization approach to ensure a broader inclusion of stakeholders’ perspective as co-designers to inform the development features and implementation of the BornFyne-PNMS platform. This process and the outcome are described in detail elsewhere [26].

The interview and FGDs explored the three phases of delay from health providers and the community’s perspective in using a digital platform to access maternal services. In addition, the team employed a typical scenario of the incident that occurred in 2016 in an urban city in Cameroon where a young lady sought care at a health facility, upon arriving at the health facility, the lady could not receive timely care partly due to inability to pay for services and other underlying delayed factors which led to her death alongside her unborn twin [23]. This scenario was used during the workshop with health providers as a mock-up scene to elucidate all possible approaches to delays that may have contributed to this outcome. This was then contextualized in a rural setting to expand on the problem taking into consideration that some of the delays in an urban setting may be exacerbated in a rural setting. This was to further inform the BornFyne-PNMS features and guide the refinement and further development of version 2.0.

### Participants for survey and interviews

The survey was designed as a door-to-door assessment administered by data collectors who are familiar with the target health district, have worked on similar projects and have conducted home visits in the past. Only women who were pregnant or had given birth before were identified for the FGDs and their men were invited including other men whose wives had recently given birth. Health providers were identified to be interviewed and community health workers (CHWs) for FGD. All age groups were represented in women from 13 to 49. The adolescent age group was included as pre-teen pregnancy was high in the French region.

### Survey sampling procedure

The districts have distinct characteristics with a mix of what can be considered rural, remote rural and semi-urban settings within the context. Based on data gathered from the districts, the estimated number of households was 18 113 in Akonolinga, 8831 in Ayos, 1100 in Bangem and 28 386 in Tiko (see Fig. 2). We determined the number of households to be surveyed based on the cost per household visit, the availability of project resources and the project timeline. We estimated the total number of households to be surveyed per district based on the estimated cost of 3000 CFA per day (<$10USD) and a maximum of 30 households per CHW per day. Thus, it was anticipated that a total of 1300 households would be surveyed in Akonolinga, 1000 in Ayos, 2000 in Tiko and 800 in Bangem.

### Interview and FGD procedure

**Figure 3:**
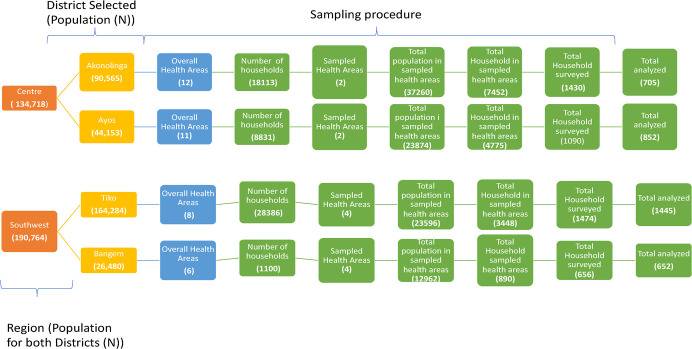
Steps in the development of BornFyne-PNMS version 2.0

Interviews were conducted with providers. Providers from a mix of private, public and confessional health facilities were identified to be interviewed. FGD was conducted with health providers during the workshop using the mock scenario. FGD was conducted for women, men and CHW. A convenient location and time were agreed upon for the FGD. Figure 3 outlined all the steps in the development process.

### Data collection and tools

#### Survey

Data collectors administered the survey, a three-point Likert scale, was employed, and respondents were asked to answer Yes, No or Not sure. The survey contained 80 questions; however, this study is focused on 12 questions including the demographic questions. The objective was to assess households with mobile phones, network coverage and PW and their perspective on using mobile phones to connect to health facilities and receive care during pregnancy. The survey lasted for 30–45 minutes, and informed consent was provided to all households. Trained data collectors who have been conducting similar surveys were employed and used a door-to-door approach to select households for the survey, and any household that refused to participate was skipped. Instead, the next household was visited until the timeline for the survey was reached or resources for the survey were exhausted.

#### Interviews

Interviews for providers explored health providers’ perspectives on the use of digital platforms to deliver care with a focus on challenges and facilitators attributed to the third delay. The questions for the survey were applied in a semi-structured format with slight modifications to understand the provider’s perspective of using digital platforms like BornFyne-PNMS to deliver reproductive maternal newborn child and adolescent health (RMNCAH) at their health facility in addition to other questions related to the three-delays model. The interviews commenced with warm-up questions to explore the provider’s role at the health facility and were followed by challenges and facilitators in delivery care to PW especially during an emergency. This was further probed with the perspective of using a digital platform to support or facilitate care during pregnancy with a focus on emergencies at their health facilities.

#### FGD

A subset of women was invited during the survey for the FGD if they’ve been pregnant and/or have delivered before. The questions for the survey were applied in a semi-structured format to understand the perspective of using digital platforms in addition to other questions related to the three-delays model. The discussion started with a warm-up question to explore women’s pregnancy and previous experiences. This was followed by a situational assessment of the current pregnancy, their challenges and facilitators in seeking and accessing care and further explored the use of a digital platform to facilitate access to health facilities. Women were asked to list possible advantages or disadvantages of using a phone to be connected to a health facility. Women were also asked to present any possible experiences or scenarios that they may have come across similar to the mock example and to identify possible intervention solutions within their context. The FGD for men was specific to men whose women participated in the FGD and were pregnant with a few additions of some men whose wives were not part of the focus group, but their wives recently gave birth. The questions for men were the same as those of the women.

For CHWs, the questions were the same with minor adaptation on looking at it from a community perspective especially as community health workers are more exposed to the household and their perspective and responses were tailored to the community perspective. In addition to informed consent, parental consent was obtained verbally for all participants below 21 years. FGD was done separately for men and women. The FGDs were used to explore women’s and households’ challenges in seeking, accessing and receiving care at health facilities during pregnancy. This was mostly tailored around key concepts of the three delays with a focus on phase 1 and 2 delays. For CHW, we explored the community’s challenges in accessing health facilities and using specific health facilities during pregnancy and the use of digital health as an intervention. In addition, workshops were conducted with health providers with a 30-minute session fashioned in a FGD format using the mock-up scenario of the incident that illustrates the ‘lady who attended the health facility but could not receive timely care and lost her life and her unborn twins’ to elucidate potential underlining delays that could have led to the incident attributable to a rural context and potential intervention solutions to address such incidence. The workshop is presented in detail in a subsequent paper. This study only captured the workshop discussions related to the identification of potential delays that could be attributed to the incident related to the mock-up scenario.

#### Equity considerations in the design and implementation of the BornFyne-PNMS platform

The design and implementation of the BornFyne-PNMS platform employed an equity focused lens PROGRESS (Place, race, occupation, gender, religion, education, socioeconomic status, social capital) plus characteristics (age, disability) [36]. Informed by the PROGRESS lens [36], we identified three major equity components in the design and implementation of a digital platform; the population (users of the platform) to be targeted, the context that the intervention is implemented(rural) and the consideration of literacy level (educational status).

##### *Population to be targeted (pregnant women and households)*.

Defining the target population is fundamental in the design of a digital platform. Population is an equity concept which in the case of the BornFyne-PNMS. PW constitute one of the primary users; therefore, gaining a deep understanding of their challenges help tailored the intervention to meet the specific needs and objectives in improving maternal health outcomes. This ensures the platform is not only effective but also responsive to the unique circumstances of PW. Designing interventions inclusive of populations experiencing vulnerability as co-designers will promote equity in access.

##### *Context of implementation*.

Commencing the interventions in rural settings is rooted in the fact that most rural settings often face unique healthcare challenges, including limited access to healthcare facilities, infrastructures and resources [37]. In addition, our assumption that an intervention designed for a rural setting can be adapted for urban areas is grounded in the understanding that rural settings typically have more limited resources and infrastructure thus, we assumed that digital intervention that works in such conditions is likely to be adaptable to urban settings with more resources and infrastructure. Urban settings have their unique challenges, including population density and potentially different healthcare infrastructure. However, starting in a rural context provides an opportunity to refine the intervention in a resource-constrained environment before scaling it to urban areas that has more resources and infrastructures. Thus, successful implementation in a rural setting can serve as a model for scaling the intervention to urban areas. Lessons learned and best practices can be applied to ensure smooth adaptation. In addition, health care providers in rural settings face unique challenges in delivering maternal care; thus, designing digital platform interventions requires engaging with health providers as co-designers and a careful understanding of their challenges to facilitate adaptation.

##### *Consideration of literacy level and populations experiencing vulnerability*.

Recognizing the challenges faced by nonliterate and populations experiencing vulnerability is essential in designing digital interventions. Designing the BornFyne-PNMS platform taking into consideration their realities and needs in mind can make it more accessible and effective. Thus, prioritizing the needs of nonliterate aligns with the principles of health equity. This ensures that those who face the greatest disparities in healthcare access are not left behind. In summary, the approach of targeting populations experiencing vulnerability and households in rural setting while considering the needs of the nonliterate population is a strategic way to promote equity in access. It allows for the development of an intervention that can potentially benefit a wider range of populations, including those with higher literacy levels in both rural and urban contexts. However, it is crucial to continually assess and adapt the BornFyne-PNMS intervention to ensure its effectiveness and relevance.

The literature has documented low literacy of women in SSA as a contributory factor to some of the delays in accessing and receiving care [1–4, 19, 21, 22]. Thus, we assumed that if the BornFyne-PNMS platform is developed taking into consideration the realities and challenges of nonliterate and vulnerable persons, it would be easily accepted by a literate group. Secondly, if an intervention is designed taking into consideration the realities of a rural setting, it would likely and easily be adapted within an urban setting. Thus, the following assumptions underpinned the development of the BornFyne-PNMS digital platform:

***Assumption 1*:** Digital intervention that is easily understood and interpreted by populations experiencing vulnerability (in this context, PW), especially a nonliterate group would be easily interpreted by the literate group taking into consideration that the literate population has an additional alternative advantage to read the intervention labels.

***Assumption II*:** Digital intervention that considers rural and remote context challenges, and/or opportunities are likely to be easily adapted to urban context (considering that urban settings have more resources and infrastructures) to facilitate acceptability and uptake.

### Quantitative data analysis

The survey was analyzed using SPSS to generate descriptive results of percentages to describe the characteristics of respondents in the household survey and their responses in using mobile phones to connect to their health facility.

#### Qualitative data analysis for interviews and FGDs

FGD and interviews were analyzed using MaxQDA software. Thematic analysis incorporates themes from the three-delays model. Data analysis was performed by two independent coders: MN and MNN. The coded elements were reviewed and compared by AF and PO for agreement. Responses were further grouped based on each category and elements that described the categories and themes from the three-delays model. The responses from community and providers perspectives were further discussed to inform further refinement of the BornFyne platform.

**Table 1:** Socio-demographic characteristics of respondents for the household survey

Demographic characteristics	Tiko (*N* = 1445)	Ayos (*N* = 852)	Akonolinga (*N* = 705)	Bangem (*N* = 652)
	*n* (%)	*n* (%)	*n* (%)	*n* (%)
Gender				
Men	362 (25.1)	404 (47.4)	436 (61.8)	132 (20.2)
Women	1083 (74.9)	448 (52.6)	269 (38.2)	520 (79.8)
Marital Status				
Married	778 (53.8)	257 (30.2)	186 (26.4)	433 (66.4)
Divorced	38 (2.6)	8 (0.9)	13 (1.8)	11 (1.7)
Single	443 (30.7)	381 (44.7)	404 (57.3)	159 (24.4)
Widowed	160 (11.1)	46 (5.4)	68 (9.6)	42 (6.4)
Prefer not to say	26 (1.8)	160 (18.8)	34 (4.8)	7 (1.1)
Education				
Primary	522 (36.1)	137 (16.1)	124 (17.6)	179 (27.5)
Secondary	580 (40.1)	359 (42.1)	297 (42.1)	242 (37.1)
High School	259 (17.9)	152 (17.8)	191 (27.1)	127 (19.5)
Bachelors	33 (2.3)	97 (11.4)	35 (5.0)	75 (11.5)
Masters	6 (0.4)	4 (0.5)	2 (0.3)	3 (0.5)
PhD	5 (0.3)	9 (1.1)	8 (1.1)	0 (0.0)
No formal education	40 (2.8)	27 (3.2)	2 (0.3)	18 (2.8)
Other	0 (0.0)	67 (7.9)	46 (6.5)	8 (1.2)

**Table 2:** Mean age and characteristics of household respondents

	Tiko *N* = 1444		Bangem *N* = 652		Ayos *N* = 852		Akonolinga *N* = 705	
	Mean	SD	Mean	SD	Mean	SD	Mean	SD
Age of survey respondents	41.27	13.56	34.88	11.58	36.46	12.23	42.67	15.11
How many people live in your household in total?	5.41	2.68	5.99	2.69	6.16	2.53	6.69	3.35
Number of males in your household	2.32	1.62	2.85	2.05	5.20	2.87	4.38	3.33
Number of females in your household	3.15	1.85	3.23	1.68	2.77	1.65	2.69	2.05
Total adolescent per household	1.43	1.52	1.59	1.54	2.86	2.02	2.85	1.96

### Ethics approval

Ethical approval was obtained from the National Ethics Board of Cameroon ref # 2022/07/1467/CE/CNERSH/SP and the University of Ottawa Social Science Ethics Board ref #H-05-22-8077. Administrative clearances were obtained from the Ministry of Public Health at the national level in Cameroon (ref. D30-1440 No. 631-3822) in collaboration with the Division for Health Operations Research (DROS) in Cameroon, the Southwest Regional Delegation of Public Health (ref. P412/MINSANTE/SWR/RDPH/CB: PF/941/618), and the Central Regional Delegation of Public Health (ref. 1393-4/AAR/MINSANTE/SG/DRSPC).

## RESULTS

### Quantitative results

In Tiko and Bangem, most of the survey respondents were women; however, in Akonolinga, mostly men responded to the survey (Tables 1 and 2). In Ayos, the number of female respondents was almost equal to the number of males as shown in Table 1. The survey was focused on understanding the household’s perspective on using mobile phones to receive care and possession of mobile phones and network coverage. Over 90% of respondents reported that they have never used a mobile phone to be connected to their health facility or health care provider. Meanwhile, over 85% are willing to use a mobile phone to communicate or connect to health facilities. Most households responded that they own a phone, but only close to 30% have a smartphone with the least reported in Akonolinga. Most households responded that they have network coverage (see Table 3). Most respondents reported that they walk to their health facility or use a bike.

**Table 3:** Household assessment of mobile phone possession and perspective of using a mobile phone to connect to a health facility

Response	Tiko *N* = 1445	Akonolinga *N* = 705	Ayos *N* = 852	Bangem *N* = 652
	*n* (%)	*n* (%)	*n* (%)	*n* (%)
Used a mobile phone to connect to a doctor or your health facility				
Yes	42 (3)	21 (3)	70 (8)	117 (18)
No	1359 (94)	396 (56)	782 (92)	528 (81)
No response	44 (3)	288 (41)	0 (0)	7 (1)
Used a mobile phone to communicate with a doctor during an emergency				
Yes	31 (2)	46 (7)	118 (14)	166 (25)
No	1382 (96)	658 (93)	734 (86)	482 (74)
No response	32 (2)	1 (0)	0 (0)	4 (1)
Willingness of participants and their household to use a mobile phone to connect with a health facility during pregnancy				
Yes	783 (54)	660 (94)	756 (89)	593 (91)
No	541 (37)	45 (6)	96 (11)	46 (7)
Not sure	84 (6)	0 (0)	0 (0)	11 (2)
No response	37 (3)	0 (0)	0 (0)	2 (0)
Willingness of participants to receive text messages for antenatal care through mobile phones				
Yes	634 (44)	669 (95)	711 (83)	637 (98)
No	716 (50)	36 (5)	119 (14)	7 (1)
Not sure	0 (0)	0 (0)	22 (3)	0 (0)
No response	95 (7)	2 (0)	0 (0)	8 (1)
Notification regarding any member of household having a phone				
Yes	1156 (80)	117 (17)	315 (37)	606 (93)
No	170 (12)	587 (83)	523 (61)	22 (3)
Not sure	3 (0)	1 (0)	14 (2)	1 (0)
No response	116 (8)	0 (0)	0 (0)	23 (4)
Notification regarding any member of household having a smart phone				
Yes	755 (52)	101 (14)	189 (22)	504 (77)
No	597 (41)	603 (86)	646 (76)	133 (20)
Not sure	0 (0)	1 (0)	17 (2)	0 (0)
No response	93 (6)	0 (0)	0 (0)	15 (2)
Participants response regarding having mobile network in their household area				
Yes	1316 (91)	569 (81)	773 (91)	631 (97)
No	58 (4)	102 (14)	31 (4)	6 (1)
Not sure	4 (0)	14 (2)	48 (6)	1 (0)
No response	67 (5)	20 (3)	0 (0)	14 (2)

Based on the assessment of mobile phone possession, Table 3 describes respondents’ perspectives on the use of mobile phones, the possibility to communicate with health care providers as well as the willingness to receive notification for ANC services. Most men possessed smart phone compared to the number of women who reported owning one.

**Figure 4:**
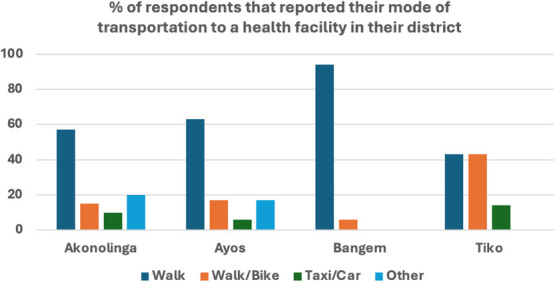
Transportation means used by respondents to reach the health facility.

Figure 4 demonstrates the means used by participants to access health facility with the majority mentioned walking as their main means of transport whether the distance is far or not. Women reported walking as their main mode of transportation to the health facility during antenatal and most of the women had much lower level of education compared to the men.

**Figure 5:**
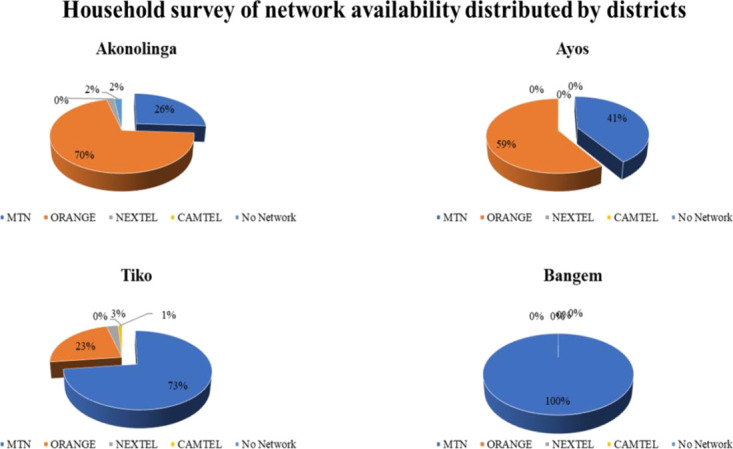
Network coverage, connectivity, and availability

Figure 5 demonstrates how the various networks are being used. For the Bangem district, participants notified MTN Network as the only network being used in the district. Participants in the Ayos district mentioned both Orange and MTN networks. But for both Tiko and Akonolinga district whereby the various participants mentioned almost all the various networks used in their district despite having higher percentages in MTN and Orange with 26% and 70% for Akonolinga and 73% and 23% in Tiko district respectively.

### Qualitative results

#### Characteristics of respondents for interviews and FGD

A total of 25 providers were interviewed across the four districts including male and female. Majority of female respondents were identified as nurses. Community health workers were mostly middle-aged 30 years and above with mostly primary and secondary education (see Table 4). PW of adolescent age group were observed more in Akonolinga as shown in Tables 3 and 4, whereas, in Tiko and Bangem, it was mostly above 20 years. We conducted an overall total of 12 FGD sessions plus four workshops fashioned after the FGD session, one per district. Four main categories of respondents were involved which include health care providers, community health workers, PW and men (see Table 5). Each of these respondents has their own understanding regarding delays in accessing and receiving prenatal services as described below.

**Table 4:** Individual distribution of respondents for FGD by district involving their demographics characteristics

Participants	District	Age	Gender	Occupation	Education
Men	Ayos	29	Male	Student nurse	Student
Men	Ayos	29	Male	Electrician	Secondary
Men	Ayos	24	Male	-	Secondary
Men	Ayos	36	Male	-	High school
Men	Akonolinga	20	Male	-	High school
Men	Akonolinga	44	Male	-	Secondary
Men	Akonolinga	44	Male	-	Secondary
Men	Akonolinga	42	Male	-	Secondary
Men	Akonolinga	20	Male	-	High school
Men	Akonolinga	21	Male	-	High school
Men	Akonolinga	50	Male	-	Primary
Men	Akonolinga	22	Male	-	High School
Men	Tiko	42	Male	Farmer	-
Men	Tiko	37	Male	Teacher	Secondary
Men	Tiko	55	Male	Farmer	-
Men	Tiko	38	Male	Trader	-
Men	Tiko	36	Male	Trader	-
Men	Tiko	30	Male	Mechanic	-
Men	Tiko	34	Male	Driver	-
Men	Tiko	28	Male	Teacher	-
Men	Bangem	39	Male	Farmer	-
Men	Bangem	32	Male	Farmer	-
Men	Bangem	48	Male	Trader	-
Men	Bangem	37	Male	Farmer	-
Men	Bangem	50	Male	Farmer	-
PW	Ayos	18	Female	Student	Secondary
PW	Ayos	23	Female	Student	University
PW	Ayos	32	Female	Nurse	High school diploma
PW	Ayos	22	Female	Student	University
PW	Ayos	24	Female	Student	University
PW	Akonolinga	35	Female	-	Secondary
PW	Akonolinga	30	Female	-	Primary
PW	Akonolinga	33	Female	-	Primary
PW	Akonolinga	16	Female	-	Secondary
PW	Akonolinga	15	Female	-	Primary
PW	Akonolinga	16	Female	-	Primary
PW	Akonolinga	30	Female	Student	University
PW	Akonolinga	17	Female	-	Primary
PW	Tiko	36	Female	Teacher (primary)	-
PW	Tiko	24	Female	Hairdresser	-
PW	Tiko	22	Female	Fashion designer	-
PW	Tiko	26	Female	Housewife and farmer	-
PW	Tiko	28	Female	Housewife	-
PW	Tiko	32	Female	Petit business	-
PW	Tiko	29	Female	Petit business	-
PW	Bangem	33	Female	Housewife and farmer	-
PW	Bangem	35	Female	Housewife and farmer	-
PW	Bangem	28	Female	Hairdresser	-
PW	Bangem	30	Female	Farmer	-
PW	Bangem	26	Female	Housewife and farmer	-
PW	Bangem	25	Female	Farmer	-

**Table 5:** Individual distribution of respondents for interviews and FGD by district involving their demographics characteristics

Participants	District	Age	Gender	Profession
FGD CHW				
CHW	Akonolinga	40	Female	CHW
CHW	Akonolinga	41	Male	CHW
CHW	Akonolinga	24	Male	CHW
CHW	Akonolinga	42	Female	CHW
CHW	Akonolinga	33	Female	CHW
CHW	Akonolinga	23	Male	CHW
CHW	Akonolinga	21	Female	CHW
CHW	Akonolinga	50	Male	CHW
CHW	Akonolinga	32	Female	CHW
CHW	Ayos	42	Male	CHW
CHW	Ayos	28	Male	CHW
CHW	Ayos	51	Male	CHW
CHW	Ayos	50	Male	CHW
CHW	Ayos	54	Male	CHW
CHW	Ayos	65	Female	CHW
CHW	Ayos	46	Male	CHW
CHW	Tiko	52	Male	CHW
CHW	Tiko	58	Female	CHW
CHW	Tiko	54	Female	CHW
CHW	Tiko	42	Female	CHW
CHW	Tiko	45	Female	CHW
CHW	Tiko	38	Female	CHW
CHW	Tiko	34	Female	CHW
CHW	Tiko	40	Female	CHW
CHW	Bangem	32	Female	CHW
CHW	Bangem	35	Female	CHW
CHW	Bangem	41	Female	CHW
CHW	Bangem	44	Male	CHW
CHW	Bangem	36	Male	CHW
Interviews				
HCP	Akonolinga	34	Female	Doctor
HCP	Akonolinga	48	Female	Nurse
HCP	Akonolinga	39	Female	Nurse
HCP	Akonolinga	29	Male	Doctor
HCP	Akonolinga	40	Female	Nurse
HCP	Ayos	31	Female	Nurse
HCP	Ayos	34	Male	Doctor
HCP	Ayos	36	Male	Nurse
HCP	Ayos	53	Female	Nurse
HCP	Ayos	50	Female	Nurse
HCP	Ayos	49	Female	Nurse
HCP	Tiko	21	Female	Midwife
HCP	Tiko	29	Female	Nurse
HCP	Tiko	37	Female	Midwife
HCP	Tiko	31	Female	Midwife
HCP	Tiko	32	Female	Midwife
HCP	Tiko	29	Female	Midwife
HCP	Tiko	36	Male	Nurse
HCP	Tiko	34	Female	Midwfe
HCP	Tiko	33	Female	Midwife
HCP	Bangem	29	Male	Doctor
HCP	Bangem	48	Female	Midwfie
HCP	Bangem	40	Female	Midwife
HCP	Bangem	38	Female	Midwife
HCP	Bangem	33	Female	Nurse

**Figure 6:**
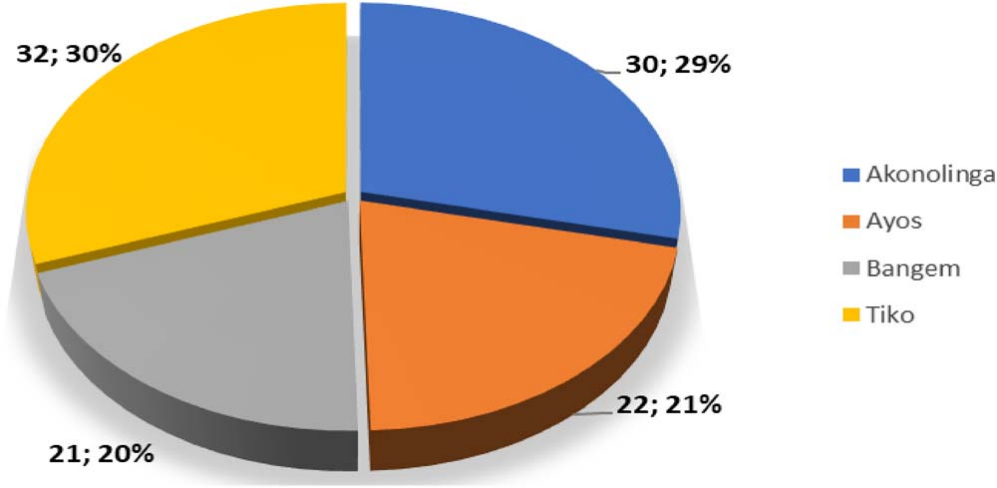
Distribution of respondents involved in the interviews and FGD distributed by district

**Figure 7:**
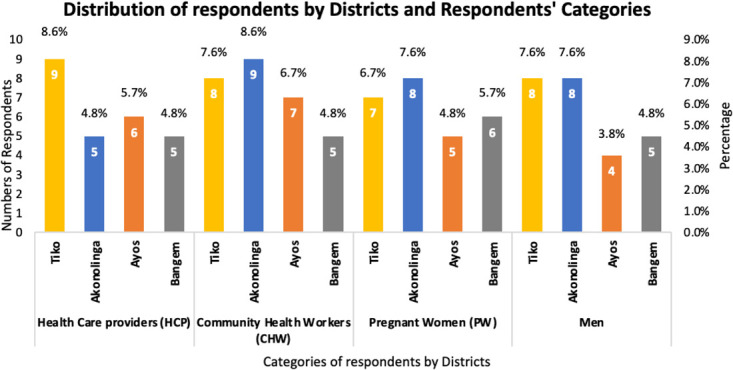
Categories of various respondents distributed by district.

Figures 6 and 7 shows the respondents distribution based on their status as either health care providers (HCP), CHW, PW and men distributed by the four districts involved in the study.

**Figure 8:**
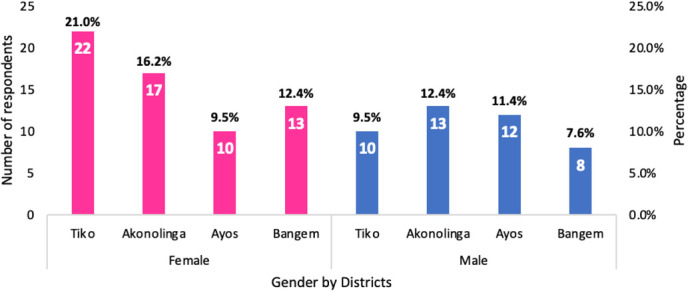
Distribution of respondents in FGD and interviews by gender and district

Basically, Fig. 8 shows the various participants’ gender in the various districts. Women had the highest with 62 (59.0%) and 43 (41%) for the men. Tiko districts had the highest number of women with 22 (21%) participants and Akonolinga had the highest number of men with 13 (12.4%).

#### ***Using the three delays to understand the contextual issues in accessing*** ANC ***in the four districts as reported by participants.***

Participants across all districts reported a wide range of delays that are interconnected and can create a complex web of barriers to accessing care by women especially in rural settings. These are categorized and described below using themes from the three delays and also illustrated in Tables 6 and 7.

#### Phase I delay: deciding to seek care.

The factors mentioned by participants across the four districts attributed to delays in seeking and receiving care are varied and can have significant impact on accessing care during pregnancy. Participants mentioned.

i) **Access to money**: Financial constraints were reported as a major barrier in seeking care. Participant struggles to afford transportation to a health care facility. This was discussed especially in relation to the issue of early pregnancy among young women below the age of 18 and was reported as a concerning health and social issue in Akonolinga district. The fact that some of the women who participated in FGD were below 18 and required parental consent for their participation highlights the gravity of the situation.

*‘When a young girl is pregnant and is impregnated by a young boy, it becomes difficult because they don’t financially have the means. To start your ANC visit here, if you don’t have at least 20–25,000cfa you will not start. When such young people arrive, incapable of even eating, they are forced to turn their backs on them. When you turn your back, it’s only the traditional/herbalist that can assist*’ [FGD Men Akonolinga/Tiko].

*‘Some have all their pregnancies without ANC visits because they don’t have money to go to the hospital because of exam fees or because the health facility is far, maybe she doesn’t have 1000 FRS to arrive at the hospital’* [FGD CHW Ayos, Akonolinga, Tiko and Bangem].

ii) **Perceived accessibility in relation to distance**: The perception of how far the health facility is from participant’s home has an influence on their health seeking behaviour and choice they made in accessing health facility. If participants believed the health facility is distant, they may delay in seeking care especially when the situation is not considered urgent.

*‘The distance is far, so when you travel on a bike and reach the hospital, you feel like you want to give birth. Even going for visits is difficult because when it rains the road is bad. I collapsed at home once because of malaria, there were no bikes, so they called the doctor, but there was no way*’ [FGD Women, Bangem/Ayos].

iii) **Weather and seasonal challenges**: Weather conditions such as heavy rains and flooding make road impassable especially in the rural areas and this has the potential to delay care for some women. Muddy and flooded roads can make it impossible for vehicles to move, and participants reported this as a potential delay in reaching health facilities.

*“Maybe it’s raining, and you are forced to enter the rain to get to your antenatal* [FGD Ayos Women].

iv) **Limited education**: Limited education can lead to lack of awareness about the importance of seeking timely care.

*‘The first issue is generally the lack of information, the women come from rural areas, and when they get here in emergency states, there is usually a lack of data’* [Akonolinga HCP].

*‘Some women are not monitored, some have no booklet and some have no documentation*’ [Akonolinga HCP].

**vi) Privacy and confidentiality**:

Participants consistently emphasized the critical role of privacy issues in hindering access to ANC. Concerns about privacy, whether related to facility-based care or the use of digital platforms, emerged as an important concern. Some women will shop around during pregnancy—that is women will travel further away from their homes for ANC clinic for various reasons despite having a health facility beside their home. This was reported especially in Tiko district. Some of the reasons as reported by participants was the way health providers manage their medical information with respect to their privacy. However, some women shop around not because of privacy, but as a way to get money from their husbands and sometimes cost of the services.

*‘To emphasize this problem, if this project was there and you took a sample of the women, you would notice that most of them are elsewhere. I’ve walked around the whole district; no one goes to facility XX. That’s the biggest issue, privacy. There’s no privacy there. As this project has come, we need to train the technicians, I think that’s the key issue. Because we can easily program the facilities for the project, and they don’t use it because of this privacy problem’* [FGD CHW, Tiko].

*‘Regarding privacy, I have a lady who told me that she confronted a nurse at the lab reception. I was angry, I told her that she was making people consult at private facilities instead of running this one. So I talked to her, and she said she had heard what I said. Once I heard that, I told her. She asked whether worked at the lab, and I said yes, that’s why I decided to meet you. She was just quiet. So this happens*’ [FGD Men Tiko].

*‘In my area, the women I have met tell me that if they go nearby, their husbands won’t give them money, so they find a way to get money from their husbands’* [FGD CHW Tiko].

#### Cultural aspects

Participants noted some cultural factors that cause delays especially in the French districts. Women do not attend ANC and would prefer to give birth at home mostly because of their husband’s decision. This was noted amongst Muslim.

*‘I somehow assisted my Muslim neighbor. The woman got pregnant, until she gave birth, to see a doctor, nothing. They refused and followed traditional ways. When the woman was in labor, they called their old woman (traditional birth attendant) to help her give birth. She spent 2 days in labour and was getting tired. Luckily, we called a doctor that was close by to help the woman give birth. When the doctor finally helps deliver the child, the doctor asked them to bring the mother and the child to the hospital for other treatment. The doctor even came with a car so I can take the mother and the child to the hospital, the father refused. Even drugs that was prescribed the father didn’t buy*.’ [Ayos/Akonolinga FGD men].

*“Yes. I know someone, he lives with a girl and every time the girl is pregnant, he prefers to go to someone that deals with herbs. At times he knows many more issue during deliver. Maybe the woman is in labor* “[Ayos FGD men].

Some of the men thought that the reminder messages to be generated for women should also be sent to their phones. They believed that some women do not keep track of details and may have limited understanding. While some believed that women are sometimes negligent.

*‘of course, I would be interested. Because I’m thinking to myself, if they only send the messages to the woman and as an example, those who are not always at home, you will not know these messages and sometimes the woman may come back and tell you the message she received, and she may have not understood which means she has not shared all the information. It’s important that I too receive the message’. [FGD men Akonolinga/Tiko].*

*‘it’s very important because at times the woman is negligent, and you need to keep her updated to put some pressure’. [FGD men Akonolinga].*

#### Phase II: Identifying and reaching the facility

##### *Actual accessibility*.

Participants reported challenges in accessing ANC and health facilities during pregnancy, including issues related to distance, cost upon arrival at the health facilities, sometimes cost exceeds expectations and they are forced to go back home without actual treatment or services. They will wait until they have enough money to get the service. They also mentioned poor roads and travel time which sometimes affect them upon arrival at the health facility.

##### *Distance*.

Participants reported distance as a barrier in accessing health facilities. They walk reasonable distances. This is illustrated in the survey where most of the participant’s mode of transportation is walking or using a bike as they do not have good roads and sometime getting a bike is also not easy coupled with bad roads during rainy seasons.

*‘Distance and cost are my main reasons. I have a farm, so I go there to cultivate, and work. Where I am, is in a suburban area, I don’t have a lot of money and we mainly do agriculture there, so I farm to sustain my family. where I am finances, cultural activities, everything disturbs us because we are in the suburbs’* [FGD Ayos Women].

##### *Cost*.

Participants reported cost as a major barrier especially as they have to pay out-of-pocket and sometimes some health facilities will prioritize payment over the services. Sometimes the actual cost will exceed their budget and they have to postpone the service for a later time.

*‘For example, financial issues are the problem. I came here twice just for lectures and went back home. I don’t have money, since the distance is not too far, I walk to attend the lecture and go back without doing lab tests*’ [FGD Women Akonolinga & Tiko].

*‘We are in an area where there is poverty, so families don’t always have money to pay, even for delivery kit that costs 6000cfa. So, there is that concern’* [FGD Akonolinga].

##### *Transportation*.

The most used mode of transportation in all four districts is walking and/or the use of motor bikes. Participants reported the difficulty when they are pregnant in using the bike and sometimes it is not easy to get the bike to certain remote areas.

*‘Also, transport means, sometimes, bikes are not easy for pregnant women so we wait until the delivery day arrives since we cannot give birth at home. We find ourselves with all difficulties’* [FGD Women Ayos/Bangem].

#### Phase III delays: receiving adequate and appropriate treatment

##### *Quality of care*.

Some participants reported dissatisfaction with the way some services are rendered to PW, concerns about inadequate patient care and negative attitudes from some health care providers and how the facilities are not taking good care of patients and focus more on money and the attitude of some health providers.

*‘Everywhere we went they were “shouting” at her saying things like “at your age you”re pregnant’ I thought they just do your job and go. I can assure you if my daughter wasn’t with me, she would have left and had an abortion. The reception at our hospitals is poor. Rejection is more common than it was at home and once she leaves the facility she begins to wonder otherwise “What am I doing with this” It’s not easy at our hospitals, my mother is a retired midwife during that time it was different, because she refused abortion cases. When you come to tell them “ I want to…” ‘they will say go away! they respond “. Economically it”s not easy’* [FGD men Akonolinga].

“*Also, when my child was ill, I took her to the clinic, they prescribed some drugs. When I got home my husband realized the drug they prescribed was not for his age. So, we took it back to the clinic, they apologized. If we gave it to that child, something bad would have happened”.* [Tiko FGD Women].

*“My sister in-law went to one clinic when she was pregnant. She took one drug they prescribed; she lost her child. It was only after that they discovered they gave her the wrong drug”* [Tiko FGD Women].

**Table 6:** Using the three-delays model to understand the contextual issues in accessing antenatal care in the four districts as reported by participants

**Phase I: deciding to seek care**			
Themes	Categories	Subcategories	Excerpts from participants
Socio-economic cultural factors	Illness factors	Recognition of complications, perceived severity	*That is more complicated outside antenatal visits. It’s during those visits that they can detect things. For example, they can find out that she doesn’t have enough blood or other things can be detected on the echography*. [Male FGD] *I was saying there are signs we can recognize, for example, when she has insomnia. For me, it’s dangerous because when you go to the hospital they would check and notice that she doesn’t have enough blood. Secondly, when a pregnant woman has constant stomach pain it’s not good. [Health provider]*
	Status of the woman	Access to money, restricted mobility, economic status, educational status	*Distance and cost are my main reasons. I have a farm, so I go there to cultivate, and work. Where I am, is in a suburban area, I don’t have a lot of money and we mainly do agriculture there, so I farm to sustain my family. Where I am finances, cultural activities, everything disturbs us because we are in the suburbs.*
	Perceived accessibility	Distance, transportation, seasonal and road conditions, cost, transportation, facility fees, medication cost, other opportunity cost	*Our distance from the home to the hospital is far, mostly bikes move along the way, and it is also very risky for us to take bikes to the hospital.*
	Perceived quality of care	Previous experience, consistent with local beliefs, staff attitudes, privacy, and confidentiality	*I too, have 2 sisters who refused to come to my health Centre for ANC. They prefer to go elsewhere. They said when labor starts, they can give birth here, but there’s no privacy at this hospital.*
**Phase II: identifying and reaching the facility**			
Themes	Categories	Subcategories	Codes and Excerpts from Participants
Actual accessibility		Distribution of location of health facilities	*It’s not easy in our hospitals. Like he said, let me take the example of a girl who got pregnant at an early age. We are in an extremely rural community, and the girl lives in a rural area 30 km from here. There may or may not be a health centre in that area, but there is one 5 to 15 km away.*
	Distance	Travel time, outcomes in transit	*The distance is far, so when you travel on a bike and reach the hospital, you feel like you want to give birth. Even going for visits is difficult because when it rains the road is bad. I collapsed at home once because of malaria. There were no bikes, so they called the doctor, there* was no way.
	Transportation	Availability of public transport	*I think the main challenge is going to the hospital because sometimes at night it is not easy to have a motorbike and also the security is not always good when it is late in the night*
	Cost	Cost exceeds expectation or ability to pay	*For example, financial issues are the problem. I came here twice just for lectures and went back home. I don’t have money., Since the distance is not too far, I walk to attend the lecture and go back without doing lab tests. Akonolinga & Tiko*
**Phase III: receiving adequate and appropriate treatment**			
Themes	Categories	Subcategories	Codes and excerpts from participants
Actual quality of care	Poorly staffed facilities	Staff numbers, competence of personnel	*You can see a young girl, surprised by her pregnancy. it all comes down to education. Let me get a bit into education because today we are in an environment where attempting to survive prevents us from taking care of our children though they may have good intentions. My youngest girl, at 16 years conceived instead of being in high school. She’s 18 and she has had 2 children. But she went back. The first time she went to the hospital, I said they should do all the tests. It wasn’t evident once there; the practitioner did not know I was her father. If she wasn’t with me, she would have run away because of the reception. Instead of the nurse doing her job, taking parameters and such, she was making remarks*
	Poorly equipped facilities	Unavailability of blood, unavailability of drugs, availability of other equipment	*They publish everywhere that blood bags are available, I donate blood. I do the calculation on what they give us, it’s over 5000cfa but to give that blood to someone who needs it, they refuse. (frustrated) Do you see where we are going? Because of the ones that came to donate freely, they gave him 5000cfa. Transmit it to give someone life!! They say ‘If you don’t have money we cannot give you. Is it normal!!? The child survived but the mother died. [*FGD Men Akonolinga]
	Inadequate management	Incorrect diagnosis and action, poor adherence to clinical guidelines, availability of clinical guidelines, job aids in accessible format,	*Getting to the hospital when she does is also another challenge because girls in that state are not received well. The reception is not tailored to their condition which is why you have a lot of infant and maternal deaths*

Adapted from the three-delays model [20]

**Table 7:** Perspective of health providers and community in using a digital platform to deliver care during pregnancy

Themes	Sub themes Community and provider’s perspectives	Supporting excerpts from participants
Cost reduction	Community perspective: Participants recognize that digital platforms can potentially reduce the cost of accessing care during pregnancy. This was related to cost saving on transportation to health facilities which can be a barrier to some women to access health services	*It will be a good thing. It will reduce the economic gap. That is, it will facilitate some things because sometimes we don’t have transport to arrive here where you’ve invited us for this discussion, but we have WhatsApp on the phone* [Men and women FGD Akonolinga, Tiko, Ayos]
	Provider perspective: Health providers also reported potential benefit in cost reduction with efficient digital platform can streamline the processes and reduce administrative costs, ultimately leading to cost savings for the health facilities	*Complimentary because using registers and other documents like hard copies has its place meanwhile the digital method is a plus and reduce costs, it makes work easier and makes work more accessible for the population. It easily connects the patient to the doctor. It is in this light that I see both methods. Complementary because a document we might have registered in a hard copy could eventually be needed for data collection. The other is to complement the shortcomings of the latter.* [Provider male Ayos]
Saves time	Community perspective: Participants reported the potential time-saving aspect of using digital platform during pregnancy by eliminating the need for long travels to health facilities, allowing pregnant women to use their time more effectively	
	Providers perspective*:* Participants recognize that digital platform can help save time in delivery care during pregnancy and lead to improved efficiency in providing care and save time on administrative tasks	*Given that we don’t have computerized systems it’s time-consuming, and waste, you have to take a lot of time to check the archives, to register or even consult a patient. The time you take to carefully fill the consultation book of a patient is time you could use to consult more patients.* [HCP Ayos, Akonolinga, Tiko, Bangem]
Emergency: Participants see the digital platform BornFyne as a facilitator during emergencies and any event of complications during pregnancy especially when they are at home	Community perspective: participants reported on the potential of using digital platform in facilitating care during emergency. Getting quick advice or health services through digital platform can save lives during emergency	*My reason is, that I used to have cramps, so before I could leave the house to go to the hospital it would be risky. I am afraid that the distance is too far for me to walk because it might cause harm to me. So I would like to call my doctor so that he will give me first aid, what to do, so I can reach the hospital. For example, if I have a lot of pain and cannot walk, I would always like to contact my doctor so he can tell me what to use and advise me on what not to do*. [Bangem FGD Women] *I also think it’s important. Suppose you live at home alone and you don’t have your doctor’s contact. If at night you have an issue, I for example I have things that often sting me in the night. In a situation where the neighbour is not around, I can contact the doctor if I have his number*. [Women FGD Akonolinga]
	Provider perspective: Providers reported the potential of digital platform to assist in emergency situations, ensuring that pregnant women receive timely and appropriate care	*It can facilitate emergency situations and complications especially in this our rural settings with poor road and difficult transport to prepare ahead of time.*. [HCP Akonolinga, Tiko, Ayos, Bangem]
Referral facilitation	Provider perspective: Providers highlighted the benefits of digital platform in facilitating referrals. This can help streamline the processes ensuring prompt and appropriate care	*It can facilitate a good number of things and permit us to prepare ahead of time to receive her and eventually see how we could refer her elsewhere in case some difficulties occur during the antenatal follow-up. It’s very important.*. [HCP Akonolinga, Tiko, Ayos, Bangem]
Quality of care	Providers perspective*:* Providers emphasize that digital platform can enhance the quality of care provided to pregnant women, improved efficiency, access to information, and better coordination of care	*Yes of course a computerized system will facilitate work given that you will just have to type in the name of the woman and all her information pops up. You can access information about somebody from anywhere and at any time.* [Female Ayos’s provider]
Communication	Providers perspective*:* Participants highlighted multifaceted advantages of using digital health platform to enhance communication and care during pregnancy such as remote consultations, emergency response, increase patient engagement, improved continuity of care, convenience. An interesting point was raised by men in relation to the importance of using mobile phones and connecting to their health facility or doctor in such a rural setting amid poverty as described by one of the participants below	*Effectively, it will be ideal. It is a very good thing. Having the number of that medical doctor and connecting to that application and explaining our situation will be very good because sometimes we don’t have food, but we have data to converse on WhatsApp*. [FGD Men Akonplinga/Bangem]
Improve patient engagement	Community perspective*:* Participants expressed the importance in remotely connecting to their provider	*I can call my doctor when I am sick and having malaria. I call him* [FDG Bangem Women]
	Provider perspective*:* Providers expressed the importance of using digital platform to enhance continuity of care, complications and emergency	*Well, it’s possible that this digital method could be a plus in taking care of and following up on pregnant women. Because it will be a good thing to know that one of our pregnant women at a given point in time is in a difficult situation, she is facing a health challenge, or she is in an early stage of labor*. [HCP Akonolinga, Tiko, Ayos, Bangem]
Remote consultation	Community perspective: Participants highlighted that using a digital platform is beneficial for addressing minor issues that require advice, reducing the stress and inconvenience of commuting. This perspective underscores the value of technology in overcoming geographical barriers and enhances access aligning to the needs and challenges of rural communities.	*It is a good innovation because at times it is good to contact the doctor or the nurse. After all, at times you may not have the means to get to the hospital. You can explain to him that you are not feeling well and ask if you can take some medication. He can prescribe, ask you to come and take and pay when you have money. If we are not in contact with the doctor, something can happen to us, we can be at home dying.* *So communication is good when there is poverty. If he can first follow up on us and if he trusts us we can later pay for the medication or come and get treatment physically.* [Women FGD Akonolinga]
	Provider perspective: Educational platform especially for adolescent girls and boys	*It’s advantageous because it permits us to solve their problems from a distance without them necessarily having to move*. [HCP Ayos, Akonolinga, Tiko, Bangem]
Community engagement	Community perspective: Some men also saw the use of digital platform as BornFyne as a platform that can engage the community and be used as a medium to communicate to the community	*That digital platform, if it can be used as community radio, for example if that platform can link the basis of the community because I think that in my community less than 30% of the population gets information from the community radio. They have no radio, so they are not connected to the information, that platform will be good if it is connected to the household, and all can listen to the messages.* [FGD Men Akonolinga, Tiko]
Educational platform especially for adolescent girls and boys	Community perspective: During FGD men were more concerned about the vulnerability of young and adolescent girl and boys and saw the digital platform as a potential platform to educate adolescent girls and boys in this technology age and time	*We understand that an application of this type can educate a mass of people. Because with Android, the phones you can’t buy, which you don’t know of this phone, your daughter can teach you and it enters easily and adapts* [Men FGD Akonolinga/Tiko] *Concerning education, we have a real problem, though this problem is present in Africa, even in the world where we say the majority of girls give birth from 16 years old. I was shocked. And I think the problem and they call it back the virginity test. I said that it was not possible. Where are we going? When we want a girl to give birth, we tell her to go and give birth in marriage and they tell us that at 16 yes the child should do a virginity test. What is that? I realized that in the details of broadcast education. Sexual education, our children discover it effectively in mobile phones. in full scale. If the application is limited at the level of education, and what is to be avoided is that I have seen in certain applications where we propose* [Men FGD Akonolinga/Tiko]
	Provider perspective: Community health workers also reported the need to educate young girls and boys with a focus on parental leadership from home	*Parents should emphasize the education of their children, especially young girls. They are vulnerable. We should prompt parents to discuss with their children, boy or girl, we don’t know where the problem can come from*. [CHW FGD Akonolinga
Data availability and storage	Provider perspective: health care providers underscore the importance of digital platform in generating and storing patient records	*It will be advantageous in that there’s no fear of losing files or documents. It permits us to store patient’s* records. [HCP Ayos]

##### *Inadequate management*.

The participants describe situations where the prioritization of financial considerations over timely interventions can lead to adverse outcomes for PW. Additionally, disrespectful treatment within healthcare facilities is highlighted, revealing instances of criticism and harsh words that undermine the dignity of PW.

*We start asking for directions on where to go because hospitals no longer take care of the sick the way they used to. If we look at the way our mothers were, things worked very well, and they were also taking care of them. We see how things unfold in the hospitals; people are suffering. Even when the woman goes to the hospital for her visits, what they ask for, firstly, is money after taking care of the woman. When this woman doesn’t have, maybe she comes from the village and may not even have anywhere to sleep or what to eat. Maybe a woman is about to give birth and they say she has a lack of blood, but there are bags of blood because she doesn’t have money for blood bags, they delay, and the woman dies*” [FGD men Akonolinga].

##### *Unavailability of equipment*.

Providers reported the lack of equipment and tools to facilitate their work especially in rural settings. However, men were more particular about the way health facilities manage the provision of blood in the blood banks. The men are curious to understand why they will donate blood freely to the blood bank and yet, if a woman needs a blood transfusion the health facility will ask the woman to pay for the same blood that was donated freely. This was cited by the men during the discussion as an incident that occurred couple of weeks before the FGD. According to the men, the woman died due to delay in receiving appropriate care related to blood transfusion.

*“They publish everywhere that blood bags are available, I donate blood. I do the calculation on what they give us, it’s about 5000cfa, but to give that blood to someone who needs it, they refuse. (frustrated). Do you see where we are going? Because of the ones that came to donate freely, they gave him 5000cfa. Transmit it to give someone life!! They say ‘If you don’t have money, we cannot give you. Is it normal!!? The child survived but the mother died”. [*FGD Men Akonolinga].

##### Perception of health providers and participants in using a digital platform to deliver care during pregnancy

Looking at the delays highlighted by participants, we further probed to explore what they think about using a digital platform to support care during pregnancy in their community. Participants listed advantages that they believed digital platforms such as BornFyne-PNMS would help address some of the challenges that they are facing. These items are described in Table 7 and grouped into themes and sub themes. Interestingly, the providers and community saw the importance from a similar perspective, while the community reported the aspect of reducing cost in commuting and saving time and emergency providers also reported aspects of emergency facilitating referrals, saving time and improving efficiency and the quality of care. This is detailed in Table 7.

With the advent of WhatsApp and mobile phones, some women and HCPs reported that they have been using WhatsApp to communicate with their patient. Thus, providers and community saw the BornFyne-PNMS project timely to them because they have been engaging with WhatsApp which indicates the need to connect through a digital platform.

#### Potential challenges reported by participants in using digital platforms in their community and considerations for the design and implementation of the BornFyne-PNMS

Women had interesting questions about using the computer to store their records during ANC. Though they saw it as good in terms of other aspects listed above in Tables 6 and 7, they also had their worries, living in a rural setting and having constant electricity they asked a few questions related to their contextual challenges and they needed some clarifications. These challenges and considerations are also described in detail elsewhere [26].

*‘It’s a good thing but at times when working with computers or electronic systems, data can disappear and if we don’t have hospital books how can we retrieve all that information?’* [Women Akonolinga/Ayos FGD].

*‘And at times your partner wants to see that book to confirm whether you are really going to the hospital. Because some provide money for you to attend these visits. We can’t completely remove the books because when we put data in the machine, there are viruses that can corrupt data’* [Women Akonolinga FGD].

*‘The problem now is, do all women in our centre come from areas covered by network? This remains the major challenge that I think needs to be checked’* [FGD Men Ayos, Bangem].

#### Applying the characteristics of the three delays from participants within the context of the BornFyne-PNMS features. A working theory of change

##### Phase I.

According to the framework, phase 1 delay which is considered deciding to seek care on the part of the individual or family in this context, the pregnant woman in question and or the household is characterized or influenced by socio-economic and cultural factors such as financial and opportunity cost, lack of access to information, women status, previous experience with the health care system, education, culture, recognition of an emergency, gender inequality, actors involved in decision-making and perceived quality of care [19]. In the context of developing the digital platform, the six graphical features on the user interface were informed by factors related to this delay phase. In addition, taking into consideration a typical scenario of a pregnant woman in a remote rural area who is nonliterate and has financial difficulties, using pictographs some barriers are eliminated as the woman who is unable to read or write can easily interpret pictographs in various context meaning. The use of a continuous educational platform where a woman can listen to educational messages helps to break social barriers and communication, the use of local dialects in communicating the messages is an important cultural attribute that brings a sense of belonging and community engagement and easy understanding and acceptance and the use of offline features will provide access on a continuous basis. A woman in need of services with limited finances can activate the system to get information from health providers which reduces commuting time and cost associated with commuting [see Table 8].

**Table 8:** Addressing elements of the three delays model within the BornFyne prenatal management system

Themes	Categories	Subcategories	Intervention features within BornFyne-PNMS version 1.0	Intervention features in BornFyne-PNMS version 2.0	Description of features and amendment based on FGD	Potential challenges informed from FGD and considerations in the implementation of BornFyne-PNMS
**Phase I: deciding to seek care**						
Socio-economic cultural factors	Illness factors	Recognition of complications, perceived severity	The use of pictographs, continuous educational platforms, local dialect	The use of pictographs, continuous educational platforms, local dialect	Women unable to read can interpret graphics and signals and activate features without necessarily dialing numbers or reading to dial. Offline features educate women and households on complications and recognize danger signs. Potential of mitigating transportation cost as a factor that may hinder the recognition of complications	Not all women possess mobile phones or smartphones and therefore may not reach out to all women. Need to engage with telecommunications
	Status of the woman	Access to money, restricted mobility, economic status, educational status	The use of pictographs, mobile phones, messages in local dialects, connected to health facilities, reminder messages and prompts	The use of pictographs, mobile phones, messages in local dialects, connected to health facilities, reminder messages and prompts	Offline features a continuous educational platform to support women and empower them, reduce social barriers through educational messages and reduce commuting time for minor issues and save cost	Potential financial challenges may still occur and equity issues with smart phone coverage. Reduce inequities of poverty and coverage barrier using telecommunication companies to expand coverage to regular phones
	Perceived accessibility	Distance, transportation, seasonal and road conditions, cost, transportation, facility fees, medication cost, other opportunity cost	Emergency feature, prompt alerts, three-way mechanism of patient/provider and transporter system	Emergency feature, prompt alerts, three-way mechanism of patient/provider and transporter app system developed to operate offline	The use of a digital platform reduces commuting, saves time and reduces the cost of transportation through teleconsultation for minor incidents, facilitate referral Women can access providers from a distance and health facilities and report complaints	Continuous education on identifying danger signs to avoid the pitfall of reliance on teleconsultation
	Perceived quality of care	Previous experience, consistent with local beliefs, staff attitudes, privacy, and confidentiality	Personalized reminder messages, prompt intervention, improved communication	Personalized reminder messages, prompt intervention, improved communication	Improve compliance and adherence of women to antenatal care, and other treatments during ANC, increase confidence and perceived privacy	Unavailability of smartphones for most women in rural areas
**Phase II: identifying and reaching the facility**						
Actual accessibility		Distribution of location of health facilities	The use of a web-based interface (PNMS) connected to the mobile application helps provide teleconsultation	The use of a web-based interface (PNMS) connected to the mobile application helps provide teleconsultation	The use of the PNMS system will help in tele consultations for minor incidents and provision of first aid advise	Unavailability of internet connection in some very remote places. Need to engage with telecommunication
	Distance	Travel time, outcomes in transit	Use of emergency feature and the three-way mechanism	Two-way interactive system to support teleconsultation. Emergency three-way mechanism	Two-way interactive system, use of transporter application offline to enhance movement during emergency	Unavailability of smartphones for most women in rural areas, need to connect with telecommunication
	Transportation	Availability of public transport	Use of teleconsultation and transporter application	Two-way interactive system to support teleconsultation. Emergency three-way mechanism	Expanded to include a transporter application accessible offline to navigate to distressed patients during emergency. Network connection with existing EmONC network in each district to facilitate transportation	Unavailability of emergency cars to transport patients during emergency/establish network connection with existing local transport system and. Network connection with existing MoPH’s EmONC network in each district to facilitate transportation
	Cost	Cost exceeds expectation or ability to pay at health facility	Platform transforms paper vouchers into electronic vouchers	Potential savings from cost of transportation can support service utilization cost (where there is paper vouchers, BornFyne transforms into e-voucher-potential saving to be directed to other services)	Teleconsultation on minor issues has potential for women and households to save money for transportation and facility fees for other services	Platform has the potential to support poor women based on the financial sustainability plan of the BornFyne-PNMS business model still to be tested
**Phase III: receiving adequate and appropriate treatment**						
Actual quality of care	Poorly staffed facilities	Staff numbers, competence of personnel	Use of prompts, alerts, integrating guidelines WHO DAK as a decision support aid and training	Use of decision support aid, tools to inform clinical decision and diagnosis, communication improvement, identification, and stratification of high-risk pregnancy, early and timely intervention	Training on the use of decision support aid, tools to inform clinical decision and diagnosis, communication improvement, identification, and stratification of high-risk pregnancy, early and timely intervention	Unwillingness to change work ethics and a need for motivation
	Poorly equipped facilities	Unavailability of equipment	Use of computers, solar chargers, solar systems to compliment unreliable power supply	Upgrading computer systems and installing PNMS as a decision support tool to enhance decision making	Health facilities require the use of computers to enter patient records and help adherence to clinical guidelines, generate reliable and actionable data to improve quality of care and save lives	Constant power supply to deliver optimal care for maternal health and use of digital platform
	Inadequate management	Incorrect diagnosis and action, poor adherence to clinical guidelines, availability of clinical guidelines, job aids in accessible format	Integrating WHO-focused antenatal guidelines	Integrating WHO digital adaptation guidelines for antenatal care into BornFyne-PNMS to improve adherence to clinical practice guidelines, with job aids in an accessible format and minimize errors	Training of providers, peer-to peer training, build capacity of health facilities and leaders to support digital transformation	Unwillingness to change work ethics. Continuous training and alignment with national policies and partnership with Ministry of Public Health to build a sustained capacity and uptake of guideline

Themes and categories from the three-delays model [20]

##### Phase II: delay in identifying and reaching medical facilities.

This is categorized under actual accessibility of facilities with reasons of physical accessibility, e.g. distance, distribution of facilities, travel time from home to the facility, cost of transportation and lack of or poor road infrastructures [19]. The use of the digital platform to connect PW and household to their health facility and/or doctors helps to reduce some of the barriers in waiting time and access to facilities in real time, and also help alert providers during an emergency. Most importantly, reducing commuting time may potentially help save cost for regular travel to health facilities for services that can be managed over the phone through teleconsultation. Establishing a transportation network of local community transporters and using a transporter application to facilitate emergencies can help reduce delays during emergencies (see Fig. 1).

##### Phase III: delay in receiving adequate and appropriate treatment due to a shortage of staff, electricity, water, medical supplies, inadequate referral system, incompetent personnel, lack of equipment and trained personnel.

This delay is critical and requires a system approach which takes into consideration the individual from the household. This does not only require the digital platform but also other aspects of the health system [19].

The use of an emergency feature provides a timely notification of emergency services and gets the health facility and health provider prepared beforehand. Despite limited staff, when a provider is aware of an emergency with notification and can assess the patient history in one shot, it helps to facilitate referrals and address some of the delays. Also, having patient record in digital format during ANC facilitates referral and improves quality of care. Connecting to local transportation and existing emergency networks through the emergency feature especially in rural areas are important ways to reduce delays and connect women and households to local transport networks during emergencies to save lives. Ensuring access to emergency transport services can be crucial for PW who need to reach healthcare facilities quickly.

Integrating the WHO DAK elements for ANC as a standard guideline enhances the quality of data collection and provides a foundation for informed and timely decision-making. This leads to adherence to clinical protocol ensures that the care provide aligns with recognized standards, enhances clinical skills and capacity and efficiency, and thus, improves on their overall performance. By leveraging the DAK guidelines, the platform and health system not only adopts best practices but also streamlines processes to more effective and standardized ANC thus reducing some of the delays in access.

Additional modifications from lessons learnt that were incorporated from user’s feedback included the need to develop an offline mobile application for health providers to enter patient records in the absence of the internet and the need to develop an offline transporter application embedded within the emergency module.

## DISCUSSIONS

Participants highlighted various delays in accessing care, including cost, transportation, quality of care, distance and digital health has the potential to address maternal mortality issues, particularly in addressing delays in accessing and receiving care in rural settings. Participants highlighted cost and distance as significant factors for Phases I and II delays in accessing care. Additionally, participants reported crucial phase III delays attributed to cost and the quality of care, particularly concerning the availability of essential equipment. In a FGD with men, a tragic incident was recounted where such delays resulted in the death of a woman. Many respondents expressed confidence in the potential of digital platforms to streamline access to healthcare, especially in emergencies. While certain delays, like the provision of blood transfusions, may not be fully addressed by digital platforms, they can still contribute to timely interventions, cost reduction for specific treatments, enabling access to alternative treatments, accurate outcome documentation and data generation for informed interventions and policies. Resolving these delays necessitates a comprehensive approach, encompassing improvements in transportation, heightened awareness and the implementation of policies aimed at enhancing the affordability and accessibility of healthcare (table 8). As maternal mortality continues to be a significant public health issue with 95% of maternal deaths occurring in SSA [38–40], achieving a significant reduction in maternal mortality requires universal access to life-saving interventions, complemented by comprehensive emergency care and overall enhancements in the quality of maternal health care [38–40].

Survey data reveals a predominant interest of using digital platform to connect to health facilities, receive reminder messages and overall interest of using digital platform during emergency, with marked district-specific variations in the possession of mobile phones and network availability. The FGDs, in turn, shed light on the complexity of the delays on accessing care, particularly emphasizing the challenges faced by women in accessing and receiving care, and the use of digital platform, cut through both survey responses and FGDs, underscoring a shared recognition of the advantages of using digital platform to facilitate access and utilization of maternal health services especially during emergency.

Our focus in rural areas using a community approach aims to target some of these challenges in rural settings where health facilities are usually distances away and sometimes no motorable roads to reach health facilities requires reasonable walking distances; thus, respondents believed that digital health would be more appealing for women in such areas given that they can do teleconsultation and reduce commuting to health facilities for very minor issues and save cost. The BornFyne-PNMS is tailored for rural settings, acknowledging challenges such as limited network connectivity and the prevalence of non-android phones among women or households. While these challenges persist, the initiative aims to address them incrementally. Collaboration with telecommunications companies, facilitated by the Ministry of Public Health, is underway to explore how the BornFyne-PNMS can be refined to facilitate the extension of coverage to households using regular phones without android phones. Also, BornFyne-PNMS U2 interface serves as an electronic health system for RMNCAH for district health systems complimenting and interoperable with the dhis2 [26, 41]. The use of the WHO DAK guidelines integrating it into the BornFyne ANC feature is an added value to the platform in supporting adherence to clinical practice and generating complete and actionable data to support other programs, e.g. malaria in pregnancy, HIV treatment adherence in pregnancy etc.

Participants highlighted the potential of mobile phones not only as communication devices but also as tools for improving healthcare access and health outcomes in resource-constrained and rural settings, especially during emergencies. Participants stressed the importance of connecting to health facilities or to a provider, noting instances when they lacked funds for care, but had data on their phones. This suggests users can alert health facilities during emergencies or distress without financial access. Such insights shape the ongoing development of BornFyne, including collaboration with telecommunication companies to ensure seamless use of the emergency feature by women and households. This connection has also facilitated collaboration between BornFyne and the existing EmONC services, exploring ways to integrate the existing EmONC network with the emergency feature for enhanced remote access and communication during emergencies.

The use of graphics seems appealing to the participants as they can easily interpret the graphics. It should be noted that these graphics were reviewed by women in the first phase and the emergency feature was refined. In this phase, there were no issues with interpretation. Participants responses implied the use of visual communication icons and visual cues are powerful means of communication, transcending language and literacy barriers especially in context where not all users can read [42, 43]. In addition, pictographs carry cultural and contextual meaning that may be understood by a wide range of users, regardless of literacy levels. Literate users can benefit from reading the information, while nonliterate users can rely on graphical comprehension. Overall, combining text and pictographs in the BornFyne-PNMS platform acknowledges the diverse literate levels and communication preferences of the target population. This can enhance the accessibility, usability and effectiveness of the BornFyne-PNMS platform in delivering essential health information and services. Thus, involving users in the design and testing process to ensure that the graphics are easily understood and culturally appropriate.

Overall, the recognition of the advantages of using digital platforms from the perspective of the community and provider underscore the potential of digital platforms to address some of the challenges and delays in accessing care during pregnancy. This can contribute to more accessible, cost-effective and efficient prenatal care services, ultimately improving maternal health outcomes in the community. In addition, men were more particular about the use of digital platform as an educational platform to enhance knowledge for adolescent given the high rate of pregnancy (Table 6). As we continue to engage stakeholders in the design and implementation of the BornFyne-PNMS platform to ensure the platform meets, the specific needs and expectations of the community and health providers alike, we have described in detail the important role of engaging stakeholder and the processes employed for the BornFyne-PNMS in another publication [26, 43].

The BornFyne PNMS platform is still under development, and the team is working to get a simple and comprehensive platform. In addition, the team is working with the health management information system in Cameroon to ensure interoperability with national health information systems. Thus, as next steps, the BornFyne team is working towards completing the integration of the WHO DAK elements, developing the interoperability layers and working with telecommunication companies through the Ministry of Public Health to expand access to the application on regular phones. The BornFyne team has engaged with the WHO digital health unit for feedback, and the BornFyne team is working closely with the Ministry of Public Health in Cameroon to realize the vision towards addressing maternal mortality.

## STRENGTH AND LIMITATION

The study employed a participatory action research approach and engaged a variety of stakeholders ranging from men and women and decision-makers. The study uses a framework and real-world scenarios to identify subtle delays that reflect contextual realities. The study engaged participants from both French and English districts, including health providers from public, private and confessional health facilities. The study uses multiple approaches in collecting data. Using the three-delays model is essential to comprehend the obstacles that women encounter in accessing maternal healthcare and establish effective interventions that can decrease maternal mortality rates. There are potential limitations in the survey responses due to reporting bias and the selection of household respondents. The digital platform has limitations in addressing some of the third delays (which requires a more system approach) but provides a comprehensive picture of possible delays. The assumptions are based on data collected during the project and the context of the study which are yet to be tested and may not apply to another context. The BornFyne-PNMS is still under development and certain features may be modified. It should be noted that this paper does not describe the detail of each feature of BornFyne and its functionality. These are described in another papers. Each feature is described in detail in subsequent papers, and the stakeholder engagement process is described in other publications [26, 43] including the development process of integrating the WHO DAK for ANC (which is also described separately in a subsequent paper). This paper only provides an overall summary of the features and process of development. In addition, Table 7 does not comprehensively describe all the functionalities aligning to each phase of delay. There are other functionalities of the application that are not listed in detail in the table and the theory of change is a working document that is updated as required.

## CONCLUSION

This study explores challenges in ANC access and introduces the BornFyne PNMS as a potential solution. The three-delays model identifies barriers, with a focus on socioeconomic factors, perceived accessibility and care quality concerns. The BornFyne-PNMS, featuring teleconsultation and educational platforms, addresses some of these challenges. The study highlights the need for context-specific adaptations, provider training and equitable access. The system’s offline features and integration efforts aim to mitigate challenges. Successful implementation requires ongoing refinement based on user feedback and alignment with community needs.

The BornFyne-PNMS deployment includes community engagement, training and practical skills building of health workers in the use of digital technologies. The establishment of an emergency transport mechanism for response to emergency cases, assessment and upgrading of the computer hardware of enrolled health facilities and support to health system managers to review and interpret the BornFyne data and interoperability with the national health management information system. In conclusion, BornFyne-PNMS offers innovation in maternal care, contingent on addressing contextual challenges through collaboration and understanding community dynamics. By addressing the social, economic and cultural factors that cause delays in accessing maternal healthcare, we can improve maternal and neonatal outcomes.

## Supplementary Material

Web_Material_oqae012
