# Supplementary material for: Developing the BornFyne prenatal management system version 2.0: a mixed method community participatory approach to digital health for reproductive maternal health
Source: Oxf Open Digit Health. 2024 Mar 7;2:oqae012. doi: 10.1093/oodh/oqae012 (PMC11932395; doi:10.1093/oodh/oqae012)
Supplement: Web_Material_oqae012 [file Web_Material_oqae012.zip › Supplemental File 1.pdf]

BornFyne-PNMS working Theory of Change

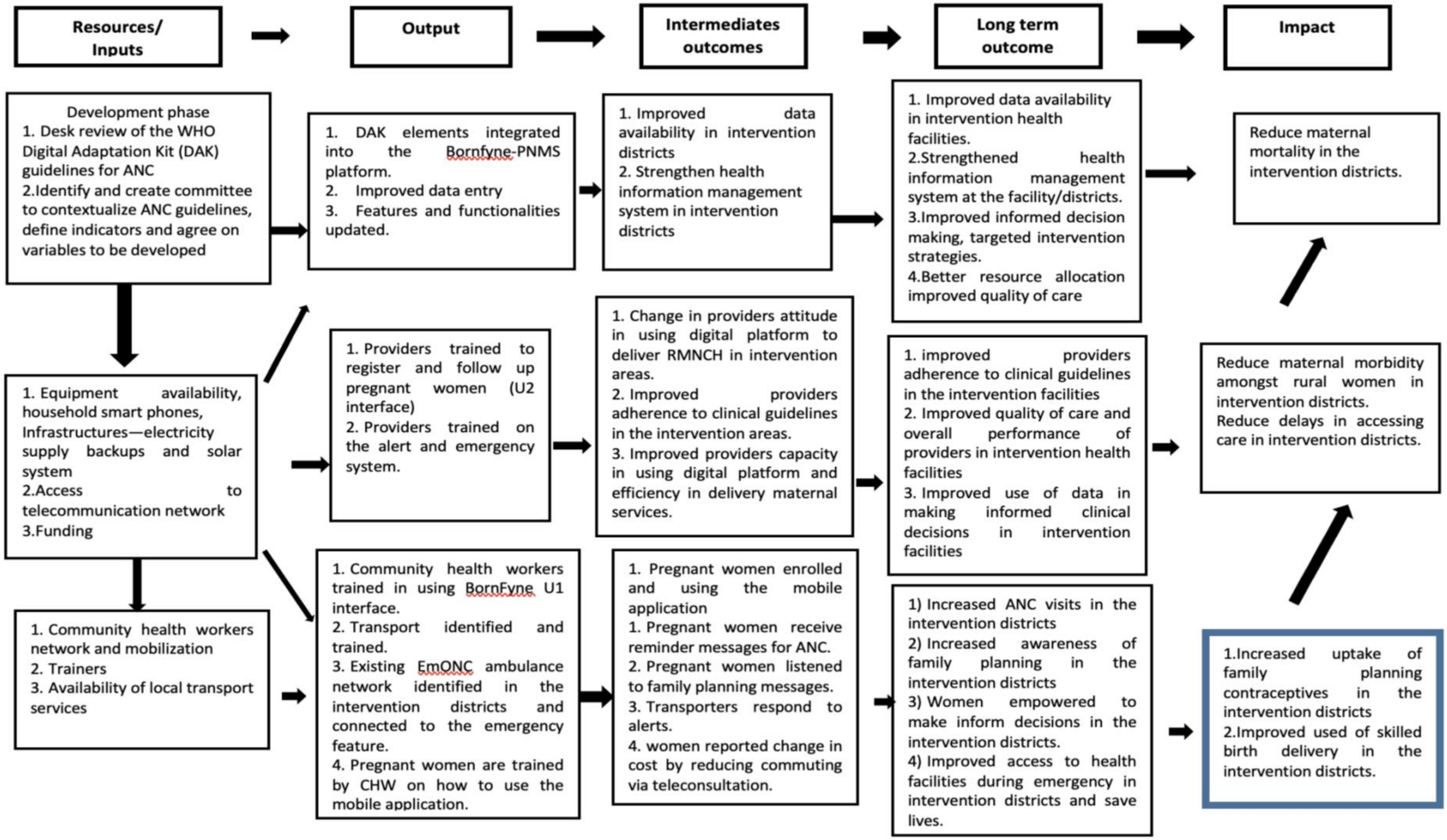

Assumptions: Ministry of Public Health is engaged at all levels to facilitate smooth implementation and to create an enabling environment and facilitate uptake. Telecommunication networks engaged to facilitate access to regular phones and expand coverage.
